# Supplementary material for: Development of a computer-aided design software for the quantitative evaluation of aesthetic damage
Source: PLoS One. 2019 Dec 18;14(12):e0226322. doi: 10.1371/journal.pone.0226322 (PMC6919621; doi:10.1371/journal.pone.0226322)
Supplement: S1 Code site — 1. (ZIP) [file pone.0226322.s001.zip › [EESC_jr][Projeto]Relatorio_07_21/reports/templates_zip/gentelella-master/vendors/jszip/documentation/examples/downloader.html]

---
title: "Mini app : Downloader"
layout: default
section: example
---

Tip : check the source of the page !

This mini application let you choose the files you want in a list, download
them, zip them and give the result to the user.

This demo requires a recent browser, see 
the howto.

### Please select your files

- Franz Kafka - The Metamorphosis.epub
- pygments.css
- jszip.js
- all.zip

pack them !
